# The Effectiveness of Mobile-Health Technologies to Improve Health Care Service Delivery Processes: A Systematic Review and Meta-Analysis

**DOI:** 10.1371/journal.pmed.1001363

**Published:** 2013-01-15

**Authors:** Caroline Free, Gemma Phillips, Louise Watson, Leandro Galli, Lambert Felix, Phil Edwards, Vikram Patel, Andy Haines

**Affiliations:** 1 Clinical Trials Unit, London School of Hygiene & Tropical Medicine, London, United Kingdom; 2 Department of Health Services Research and Policy, London School of Hygiene & Tropical Medicine, London, United Kingdom; 3 Department of Population Health, London School of Hygiene & Tropical Medicine, London, United Kingdom; 4 Warwick University, Coventry, United Kingdom; 5 Department of Primary Care and Public Health, Imperial College, London, United Kingdom; London School of Economics, United Kingdom

## Abstract

**Background:**

Mobile health interventions could have beneficial effects on health care delivery processes. We aimed to conduct a systematic review of controlled trials of mobile technology interventions to improve health care delivery processes.

**Methods and Findings:**

We searched for all controlled trials of mobile technology based health interventions using MEDLINE, EMBASE, PsycINFO, Global Health, Web of Science, Cochrane Library, UK NHS HTA (Jan 1990–Sept 2010). Two authors independently extracted data on allocation concealment, allocation sequence, blinding, completeness of follow-up, and measures of effect. We calculated effect estimates and we used random effects meta-analysis to give pooled estimates.

We identified 42 trials. None of the trials had low risk of bias. Seven trials of health care provider support reported 25 outcomes regarding appropriate disease management, of which 11 showed statistically significant benefits. One trial reported a statistically significant improvement in nurse/surgeon communication using mobile phones. Two trials reported statistically significant reductions in correct diagnoses using mobile technology photos compared to gold standard. The pooled effect on appointment attendance using text message (short message service or SMS) reminders versus no reminder was increased, with a relative risk (RR) of 1.06 (95% CI 1.05–1.07, *I* ^2^ = 6%). The pooled effects on the number of cancelled appointments was not significantly increased RR 1.08 (95% CI 0.89–1.30). There was no difference in attendance using SMS reminders versus other reminders (RR 0.98, 95% CI 0.94–1.02, respectively). To address the limitation of the older search, we also reviewed more recent literature.

**Conclusions:**

The results for health care provider support interventions on diagnosis and management outcomes are generally consistent with modest benefits. Trials using mobile technology-based photos reported reductions in correct diagnoses when compared to the gold standard. SMS appointment reminders have modest benefits and may be appropriate for implementation. High quality trials measuring clinical outcomes are needed.

*Please see later in the article for the Editors' Summary*

## Abstract 2

Caroline Free and colleagues systematically review controlled trials of mobile technology interventions to improve health care delivery processes and show that current interventions give only modest benefits and that high-quality trials measuring clinical outcomes are needed.

## Editors’ Summary

**Background:**

Over the past few decades, computing and communication technologies have changed dramatically. Bulky, slow computers have been replaced by portable devices that can complete increasingly complex tasks in less and less time. Similarly, landlines have been replaced by mobile phones and other mobile communication technologies that can connect people anytime and anywhere, and that can transmit text messages (short message service; SMS), photographs, and data at the touch of a button. These advances have led to the development of mobile-health (mHealth)—the use of mobile computing and communication technologies in health care and public health. mHealth has many applications. It can be used to facilitate data collection and to encourage health-care consumers to adopt healthy lifestyles or to self-manage chronic conditions. It can also be used to improve health-care service delivery processes by targeting health-care providers or communication between these providers and their patients. So, for example, mobile technologies can be used to provide clinical management support in settings where there are no specialist clinicians, and they can be used to send patients test results and timely reminders of appointments.

**Why Was This Study Done?:**

Many experts believe that mHealth interventions could greatly improve health-care delivery processes, particularly in resource-poor settings. The results of several controlled trials (studies that compare the outcomes of people who do or do not receive an intervention) of mHealth interventions designed to improve health-care delivery processes have been published. However, these data have not been comprehensively reviewed, and the effectiveness of this type of mHealth intervention has not been quantified. Here, the researchers rectify this situation by undertaking a systematic review and meta-analysis of controlled trials of mobile technology-based interventions designed to improve health-care service delivery processes. A systematic review is a study that uses predefined criteria to identify all the research on a given topic; a meta-analysis is a statistical approach that is used to pool the results of several independent studies.

**What Did the Researchers Do and Find?:**

The researchers identified 42 controlled trials that investigated mobile technology-based interventions designed to improve health-care service delivery processes. None of the trials were of high quality—many had methodological problems likely to affect the accuracy of their findings—and nearly all were undertaken in high-income countries. Thirty-two of the trials tested interventions directed at health-care providers. Of these trials, seven investigated interventions providing health-care provider education, 18 investigated interventions supporting clinical diagnosis and treatment, and seven investigated interventions to facilitate communication between health-care providers. Several of the trials reported that the tested intervention led to statistically significant improvements (improvements unlikely to have happened by chance) in outcomes related to disease management. However, two trials that used mobile phones to transmit photos to off-site clinicians for diagnosis reported significant reductions in correct diagnoses compared to diagnosis by an on-site specialist. Ten of the 42 trials investigated interventions targeting communication between health-care providers and patients. Eight of these trials investigated SMS-based appointment reminders. Meta-analyses of the results of these trials indicated that using SMS appointment reminders significantly but modestly increased patient attendance compared to no reminders. However, SMS reminders were no more effective than postal or phone call reminders, and texting reminders to patients who persistently missed appointments did not significantly change the number of cancelled appointments.

**What Do These Findings Mean?:**

These findings indicate that some mHealth interventions designed to improve health-care service delivery processes are modestly effective, but they also highlight the need for more trials of these interventions. Specifically, these findings show that although some interventions designed to provide support for health-care providers modestly improved some aspects of clinical diagnosis and management, other interventions had deleterious effects—most notably, the use of mobile technology–based photos for diagnosis. In terms of mHealth interventions targeting communication between health-care providers and patients, the finding that SMS appointment reminders have modest benefits suggests that implementation of this intervention should be considered, at least in high-income settings. However, the researchers stress that more trials are needed to robustly establish the ability of mobile technology-based interventions to improve health-care delivery processes. These trials need to be of high quality, they should be undertaken in resource-limited settings as well as in high-income countries, and, ideally, they should consider interventions that combine mHealth and conventional approaches.

**Additional Information:**

Please access these Web sites via the online version of this summary at http://dx.doi.org/10.1371/journal.pmed.1001363.

- A related *PLOS Medicine* Research Article by Free et al. investigates the effectiveness of mHealth technology-based health behavior change and disease management interventions for health-care consumers
- Wikipedia has a page on mHealth (note: Wikipedia is a free online encyclopedia that anyone can edit; available in several languages)
- mHealth: New horizons for health through mobile technologies is a global survey of mHealth prepared by the World Health Organization’s Global Observatory for eHealth (eHealth is health-care practice supported by electronic processes and communication)
- The mHealth in Low-Resource Settings website, which is maintained by the Netherlands Royal Tropical Institute, provides information on the current use, potential, and limitations of mHealth in low-resource settings
- The US National Institutes of Health Fogarty International Center provides links to resources and information about mHealth

## Introduction

Mobile health, the use of mobile computing and communication technologies in health care and public health, is a rapidly expanding area within e-health. There is considerable enthusiasm for mobile-health interventions and it has been argued that there is huge potential for mobile-health interventions to have beneficial effects on health and health service delivery processes, especially in resource-poor settings [1].

Mobile technologies include mobile phones; personal digital assistants (PDA) and PDA phones (e.g., BlackBerry, Palm Pilot); Smartphones (e.g., iphone); enterprise digital assistants (EDA); portable media players (i.e., MP3-players and MP4-players, e.g., ipod); handheld video-game consoles (e.g., Playstation Portable (PSP), Nintendo DS); and handheld and ultra-portable computers such as tablet PCs (e.g., ipad and Smartbooks).

These devices have a range of functions from mobile cellular communication using text messages (SMS), photos and video (MMS), telephone, and World Wide Web access, to multimedia playback and software application support. Technological advances and improved computer processing power mean that single mobile devices such as smart phones and PDA phones are increasingly capable of high level performance in many or all of these functions.

Mobile health interventions designed to improve health care service delivery processes have been used to provide support and services to health care providers (such as education, support in diagnosis or patient management) or target communication between health care services and consumers (such as appointment reminders and test result notification).

The features of mobile technologies that may make them particularly appropriate for improving health care service delivery processes relate to their popularity, their mobility, and their technological capabilities. The popularity of mobile technologies has led to high and increasing ownership of mobile technologies, which means interventions can be delivered to large numbers of people. In 2009, more than two-thirds of the world's population owned a mobile phone and 4.2 trillion text messages were sent [2]. In many high-income countries, the number of mobile phone subscriptions outstrips the population [3]. In low-income countries, mobile communication technology is the fastest growing sector of the communications industry and geographical coverage is high [4]–[7].

The mobility and popularity of mobile technologies means that many people carry their mobile phone with them wherever they go. This allows temporal synchronisation of the intervention delivery and allows the intervention to claim people's attention when it is most relevant. For example, health care consumers can be sent appointment reminders that arrive the day before and/or morning of their appointment. Real-time (synchronous) communication also allows interventions to be accessed or delivered within the relevant context, i.e., the intervention can be delivered and accessed at any time and wherever it is needed. For example. at the time health care providers see a patient, they can access a management support system providing information and protocols for management decisions to whomever requires them. This application could be particularly relevant for providing clinical management support in settings where there is no senior or specialist health care provider support or where there is no such support at night or at weekends. As mobile technologies can be transported wherever one goes, interventions are convenient and easy to access.

The technological capabilities of mobile technologies are continuing to advance at a high pace. Current technological capabilities allow low cost interventions. There are potential economies of scale as it is technically easy to deliver interventions to large populations (for example, mobile technology applications can easily be downloaded and automated systems can deliver text messages to large numbers of people at low cost). The technological features that have been used for health interventions include text messages (SMS), software applications, and multiple media (SMS, photos) interventions. The technology allows interventions to be personalised and interactive.

In this rapidly changing field, existing systematic reviews of mobile-health (M-health) interventions to improve health care service processes require updating [8]. Existing reviews have focussed on specific topics. A review of randomised controlled trials of text message reminders for appointments found small benefits and a review of the effect of test notification by text message found insufficient evidence to determine if there were benefits [9],[10]. Rapid advances in technology mean that it is now less relevant to conduct reviews focussing on specific devices (e.g., PDAs or hand-held computers [11]). Specific devices become outdated but their functions (e.g., application software) are now available on newer devices (e.g., SMART phones).

A current overview of the evidence for all mobile technology interventions evaluated in controlled trials to improve health care processes is lacking.

This systematic review aimed to quantify the effectiveness of mobile technology based interventions delivered to health care providers or to support health care services, on any health or health care service outcome.

## Methods

We adhered to our published plan of investigation as outlined in the study protocol [12].

Participants were men and women of any age. We included all controlled trials using any mobile technology interventions (mobile phones; PDAs and PDA phones [e.g., BlackBerry, Palm Pilot]; Smartphones [e.g., the iphone]; enterprise digital assistants [EDA]; portable media players [i.e., MP3-players and MP4-players, e.g., ipod]; handheld video-game consoles [e.g., Playstation Portable (PSP), Nintendo DS]; and handheld and ultra-portable computers such as tablet PCs [e.g., the ipad] and Smartbooks) to improve or promote health or health service use or quality. Trials were included regardless of publication status or language.

We only included studies in which the mobile electronic device is the stated intervention under evaluation, i.e., we excluded studies evaluating mixed mobile electronic device and non-mobile electronic device interventions such as an intervention involving face-to-face educational sessions with a software application educational intervention compared to a control group receiving paper-based information only. We excluded general videos, unless authors stated they were specifically designed to be viewed on mobile technologies. Internet interventions that were not specifically designed for mobile technologies were outside the scope of this review.

The interventions in trials meeting the inclusion criteria and aiming to improve health care delivery process are reported herein. Other trials identified are reported elsewhere [13]. No trial was excluded from the review based on the type of health or health care service targeted, but trials not directed at health care service delivery were included in one of two papers reported elsewhere, one covering behaviour change interventions and self-management of diseases for health consumers and the second, data collection [13]. Trials involving appointment reminders are included in this paper but those involving broader behavioural support are reported elsewhere [13].

The trials with interventions aiming to improve health care delivery processes were then categorised into two groups: those directed to health care providers or those involving communication between health care services and health care consumers (e.g., appointment reminders, test result notification). Interventions for health care providers were then subcategorized according to their purpose: education, diagnosis and management, and communication between health care providers. Interventions involving health care service communication to consumers were subcategorized according to their purpose: appointment reminders and test result notification. Primary outcomes were defined as any objective measure of health, health service delivery, or use. Secondary outcomes were defined as self-reported outcomes related to health behaviours, disease management, health service delivery or use, and cognitive outcomes. Outcomes reported for any length of follow-up were included.

We searched the following electronic bibliographic databases MEDLINE, EMBASE, PsycINFO, Global Health, The Cochrane Library (Cochrane Database of Systematic Reviews, Cochrane Central Register of Controlled Trials [CENTRAL], Cochrane Methodology Register), NHS Health Technology Assessment Database, and Web of Science (science and social science citation index) from 1990 to Sept 2010 and the reference lists of included trials. The list of subheadings (MeSH) and textwords used in the search strategy can be found in Text S1. All of these terms were combined with the Cochrane Library MEDLINE filter for controlled trials of interventions.

Two reviewers independently scanned the electronic records to identify potentially eligible trials.

Two reviewers independently extracted data on number of randomised participants, intervention, intervention components, sequence generation, allocation concealment, blinding of outcome assessors, completeness of follow-up, evidence of selective outcome reporting, and any other potential sources of bias on measures of effect using a standardised data extraction form. The authors were not blinded to authorship, journal of publication, or the trial results. All discrepancies were agreed upon by discussion with a third reviewer. Risk of bias was assessed according to the criteria outlined by the International Cochrane Collaboration [14]. We assessed blinding of outcome assessors and data analysts and we used a cut off of 90% complete follow-up for low risk of bias for completeness of follow-up. We contacted study authors for additional information about the included studies, or for clarification of the study methods as required.

All analyses were conducted in STATA v 11. We calculated risk ratios and standard mean differences. We used random effects meta-analysis to give pooled estimates. We examined heterogeneity visually by examining the forest plots and statistically using both the χ^2^ test and the *I* ^2^ statistic. We assessed evidence of publication bias using Funnel plots.

## Results

**Figure 1:**
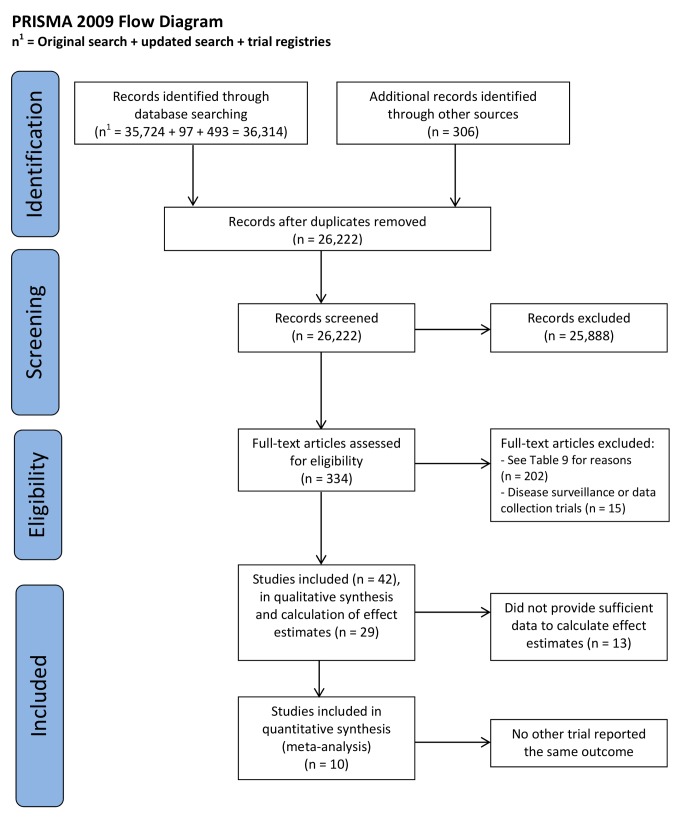
PRISMA 2009 flow diagram.

The combined search strategies identified 26,221 electronic records; these were screened for eligibility, and the full texts of 334 potentially eligible reports were obtained for further assessment (Figure 1). Out of the 334 potentially eligible reports, 42 met the study inclusion criteria and were directed at improving health care service delivery. There were 32 trials of interventions designed to support health care providers and ten trials of interventions targeting communication between health services and health care consumers.

### Characteristics of Studies

#### Heath care provider support

The 32 trials included 5,323 participants. Samples ranged from 14 to 1,874 participants. There were 15 randomised controlled trials with parallel groups, six randomised cross over trials, three cluster randomised controlled trials, and eight non-randomised controlled trials. Seven were trials of health care provider education [15]–[21] (Table 1), 18 were trials of interventions supporting clinical diagnosis and management [22]–[39] (guidelines, protocols, decision support systems; Table 2), and seven were trials of interventions designed to facilitate verbal or data communication between health care providers for clinical/patient management [40]–[46] (Table 3). Of these one used mobile technologies for verbal communication and seven communicated images. All the trials were conducted in high-income countries.

**Table 1:** Interventions applied for medical education.

Study	Study Design, Country, Device, Media	Participants	Aims	Intervention	Comparator
**Farrell 2008 ** [**15**]	Other; *Country*: Australia; *Device*: PDA; *Media*: Application software	76 medical-surgical second years. Mean age: Control = 20 y; Intervention = 22.5 y	To investigate whether the use of PDAs enhanced nursing students' pharmacological knowledge during clinical practice in the medical-surgical area.	Nursing students in the experimental group each were provided with a PDA loaded with the MIMS for PDAs, a drug reference guide for Australian professionals, as well as two Excel documents, a student appraisal tool, and a medical-surgical nursing skills checklist. Duration: 3 wk	The control group completed a similar clinical placement without access to a PDA.
**Goldsworthy 2006 ** [**16**]	Parallel group RCT; *Country*: Canada; *Device*: PDA; *Media*: Application software	36 second-year baccalaureate nursing students in one of two community acute care hospitals.	To determine if the use of a PDA influences the students' preparedness for the safe administration of medications and enhances the students' self-efficacy.	The PDA was loaded with software including a laboratory and diagnostic reference, a drug reference book, and a medical-surgical procedure resource from Elsevier Publishing.	The control groups were given access to paper versions of the same resources that were given to the intervention group.
**Leung 2003 ** [**17**]	Parallel group RCT; *Country*: Hong Kong; *Device*: PDA; *Media*: Custom software	114 fourth-year undergraduates in their senior clerkship. Mean age: Control = 22.4 y (SD: 1.1); Intervention = 22.5 y (SD: 1.3).	To test whether providing medical students with a handheld computer clinical decision support tool could improve learning in evidence based medicine.	The intervention is InfoRetriever software loaded onto a PDA. It contains seven evidence databases clinical decision rules and practice guidelines, risk calculators, and basic information on drugs. This software as well as a digital version of the pocket card were loaded onto a PDA. Duration: 16 wk.	The control group received a pocket card containing guidelines such as the evidence-based decision-making cycle, levels, and sources of evidence, and abbreviated guidelines on appraising the relevance and validity of articles about diagnostic tests, prognosis, treatment, and practice guidelines. The card was designed to remind and prompt students to apply evidence-based medicine techniques in their clinical learning.
**Mcleod 2006 ** [**18**]	Parallel group RCT; *Country*: USA; *Device*: PDA; *Media*: Custom software	52 first-year medical residents rotating on a general medical hospital service.	To design, implement, and evaluate the educational effectiveness of a PDA-based geriatric assessment tool among internal medical students.	A geriatric assessment tool was developed based on an 8-module course. First-year residents who were PDA users were randomised to receive the geriatric assessment tool software on their PDA. Performance on a pre/post test and tabulation of geriatric functional issues identified on hospital dismissal summaries were the outcomes measured. Duration: 1 y	Participants in the control group owned a PDA, but did not receive the geriatric assessment tool software on the device. They had web-based access to the geriatric assessment tool, as did the intervention group.
**Mcleod 2009 ** [**19**]	Parallel group RCT; *Country*: USA; *Device*: PDA phone; *Media*: Application software	72 first-year residents rotating on the primary care internal medicine and geriatrics hospital service.	To evaluate the educational effectiveness of a PDA-based GAT.	At the outset of their rotations, all residents received instruction focusing on geriatric functional assessment. Eight topics were presented: ADL, IADL, cognition, mobility, depression, delirium, malnutrition, and risk of adverse drug events. Functional assessment measures for the 8 lecture modules were incorporated into a web-based application and the intervention group had this application loaded onto their PDAs. Duration: 1 mo	Control group had access to this information, but not on PDA.
**Strayer 2010 ** [**20**]	Parallel group RCT; *Country*: USA; *Device*: PDA; *Media*: Application software	122 third-year medical students.	To determine if a PDA-based smoking cessation counselling tool can improve medical student smoking cessation counselling.	All students underwent a workshop on motivational interviewing. The intervention group received a paper-based summary of motivational interviewing techniques relating to SCC following the workshop, and also E-SMOKE-I.T. Software loaded onto their PDA. The software helps users determine a patient's stage of change, provides scripted motivational interviews targeted to their stage, and makes relevant health behaviour and stage-based interventions immediately accessible. A smoking cessation counselling assessment tool was developed and validated to assess students' expertise. Duration: 4 wk	The control group received a paper-based summary of motivational interviewing techniques related to smoking cessation counselling following the workshop.
**Tempelhof 2009 ** [**21**]	Parallel group RCT; *Country*: USA; *Device*: Portable media player; *Media*: MP4/video	30 residents willing to attend five prescheduled midday conferences.	To assess whether medical resident participation in educational conferences using mobile iPod technology enhances both medical knowledge and accessibility when compared to residents only participating in person.	Residents were required to be absent from the five lunchtime conferences, to download the conferences from the Duke University iTunes website, transfer to the iPod, and listen to them. Duration: 1 mo	Control group were required to attend a specific series of five, 1-hour midday conferences. These attendees were allowed to leave the conference for personal or patient care issues.

ADL, activities of daily living; IADL, instrumental activities of daily living; MIMS, Monthly Index of Medical Specialties; RCT, randomized controlled trial.

**Table 2:** Interventions applied for clinical diagnosis and management.

Study	Study Design, Country, Device, Media	Participants	Aims	Intervention	Comparator
**Berner 2006 ** [**22**]	Parallel group RCT; *Country*: USA; *Device*: PDA phone; *Media*: Application software	66 internal medicine residents assigned to an urban university-based, resident-staffed clinic. Mean age: Control = 28.6 y (SD 2.28), 72% male; Intervention = 27.4 y (SD 2.18), 74% male	To determine whether clinicians provided with a CDSS that provides recommendations for risk assessment and treatment prescribe NSAID more safely than clinicians without that support. What is the impact of the CDSS on participants' gathering key risk factor data?	Medical residents received a PDA-based CDSS suite. This included a prediction rule for NSAID-related gastrointestinal risk assessment and treatment recommendations. Unannounced standardised patients trained to portray musculoskeletal symptoms presented to intervention group. Safety outcomes were assessed from the prescriptions given to standardised patents and judged as safe or unsafe.	Control group did not receive the prediction rule for NSAID-related gastrointestinal risk assessment and treatment recommendations.
**Blaivas 2005 ** [**23**]	Crossover trial; *Country*: USA; *Device*: Mobile telephone; *Media*: Picture	Credentialed sonologists	To compare high-resolution thermal printer ultrasound images and images recorded and transmitted via commercial camera cell phones.	2 credentialed emergency sonologists with extensive ultrasound experience were asked to evaluate images on a cell phone. A limited clinical vignette was then read to each of the 2 reviewers describing patient complaint and area of the body being scanned. Reviewers were also asked if any pathology was seen in the image, if any measurements were present and what they were, and if a diagnosis could be given and to list major visible structures. Finally, each reviewer was asked if the image being viewed was either suboptimal for review or contained image artefacts other than expected from ultrasound.	2 wk later the process was repeated for the thermal printer images, or originals.
**Bürkle 2008 ** [**24**]	Parallel group RCT; *Country*: Germany; *Device*: Tablet PC; *Media*: Custom software	Intensive care nurses	To evaluate a computer-based scoring tool in an ICU. User satisfaction, time needed to score a patient and workflow change were assessed, and scores generated manually and by computer were compared.	CORE10TISS, TISS28, TISS76, APACHE2, and SAPS2 are scores that must be calculated daily for each patient in ICU. Prior to the intervention all results were calculated manually; this intervention introduces a method of scoring using a tablet PC. An evaluation protocol was developed to assess workflow analysis, time series questionnaire technology, time consumption, and score values.	Scoring was performed and timed manually.
**Coopmans 2008 ** [**26**]	Crossover RCT; *Country*: USA; *Device*: PDA; *Media*: Custom software	4 certified registered nurse anaesthetists (CRNA)	To evaluate a method for examining the effect of CADM on the accuracy and speed of problem solving during simulated critical patient care events.	A PDA was pre-programmed with a catalogue of common and uncommon clinical events that provided a protocol-driven, interventional approach to management. Two patient care scenarios were developed for this study. Within each scenario, the simulated patient's problem or condition, if left unattended, could lead to a critical incident. scenarios. CRNA performance with and without CADM technology was evaluated.	Control participants were instructed to use their own knowledge, beliefs, customary approaches, and experiences.
**Greenfield 2007 ** [**27**]	Non-randomised cross-sectional; *Country*: USA; Device: PDA; *Media*: Application Software	87 undergraduate nurses	To determine whether nursing medication errors could be reduced and nursing care provided more efficiently using PDA technology	Students in the intervention group used PDAs equipped with a drug program created for health care providers, which is continually reviewed and updated with more than 3,500 brand and generic drugs	Students in the control group could use textbooks and reference books found on most clinical units, such as medical-surgical nursing textbooks, pharmacology textbooks, a drug reference guide, and a calculator.
**Greiver 2005 ** [**28**]	Parallel group RCT; *Country*: Canada; *Device*: PDA; *Media*: Application software	18 30–75 y olds presenting to see their family physicians with symptoms judged to be possible new-onset angina	To determine the effectiveness of a PDA software application to help family physicians diagnose angina among patients with chest pain	Physicians in the intervention arm received Palm OS-based hand-held computers loaded with the angina software. Monthly reminders were sent to all physicians (control and intervention) to maximize patient recruitment and to minimize recall bias. Duration: 7 mo	Physicians in the control group were instructed to continue to manage patients presenting with chest pain in their normal manner.
**Jayaraman 2008 ** [**29**]	3 arm parallel group RCT; *Country*: New Zealand; *Device*: Mobile phone; *Media*: photos	30 health care providers from primary care	To determine the effectiveness of adding photos to clinical history on diagnostic confidence with (1) photos viewed on mobile phones and (2)photos viewed via email.	Health care providers were provided with 10 clinical case histories and allocated to one of 2 interventions either to view photos of these clinical conditions on a mobile phone or to view photos on a CD ROM (to simulate the type of photos that would be viewed by email on a computer).	The control group had access to the case histories only
**Lee 2009 ** [**30**]	Cluster RCT; *Country*: USA; *Device*: PDA; *Media*: Custom software	1,874 patients documented by 29 nurses enrolled in a masters program. Mean age: Control = 47.16 y (SD: 16.95), 42.5% male; Intervention = 47.8 y (SD: 17.88), 41.5% male	To compare the proportion of obesity-related diagnoses in clinical encounters documented by nurses using a PDA-based log with and without obesity decision support features.	The intervention group had on their PDAs a clinical decision support system for obesity management. On the basis of the results of screening, the clinical decision support system generates an obesity-related diagnosis, and nurses documented the patients' weight management goal.	The control group filled in a clinical log that supports entering of height and weight, selection of an obesity related diagnoses from a pick-list of diagnoses for “weight-related condition”; and selection of plan of care items from pick-lists.
**Mclaughlin 2010 ** [**31**]	Cluster RCT; *Country*: USA; *Device*: PDA; *Media*: Application Software	1,662 patients between 3 and 18 y of age being seen for a well visit were eligible for the study	To determine if (a) clinic staff will accept and use new interventions for BP screening in children and (b) if simple in-office interventions such as an abridged normative BP table in the medical record or provision of a BP percentile as part of the vital signs can improve physician recognition of abnormally high BP.	All 3 study groups (2 intervention, 1 control) followed the same standard of having a nurse/medical assistant take and record a seated BP measurement at the beginning of all well visits starting at age 3 y. BP Group: This group used a condensed version of the current normative BP tables. The condensed chart was printed on 4×6-in self-adhesive labels. Those responsible for checking inpatients were instructed to add a gender-specific BP sticker in the upper LH corner of the patient's growth chart prior to giving the record to the physician. PDA Group: This group used a PDA application that calculated a BP percentile or percentile range for each BP value entered. The PDA application allowed the nurse/medical assistant to enter the patient's age, gender, height, weight, and BPs. Information was printed on a receipt that was attached to the examination form in the medical record.	The control group received no intervention; clinicians continued their preferred individual practice.
**Momtahan 2007 ** [**32**]	Non-randomised parallel group trial; *Country*: Canada; *Device*: PDA; *Media*: Custom software	16 nursing coordinators	To demonstrate the viability and value of implementing a cardiac decision support tool on PDAs to deliver standardized care to cardiac patients using a human factors approach to the design.	The intervention was a decision support tool on a PDA for cardiac tele-triage/tele-consultation. In the intervention group, NCs used the tele-form on the PDA when they received chest pain calls from patients over the 3-mo period. Duration: 3 mo	In the control group NCs used the paper-based teleform when they received chest pain calls from patients.
**Price 2005 ** [**33**]	Parallel group RCT; *Country*: Canada; *Device*: PDA; *Media*: Application software	79 patients, not pregnant, aged 18 y or older, and able to provide informed consent. Control = 75% male; Intervention = 50% male.	To examine whether Palm Prevention improved adherence to five preventive measures in primary care.	Palm Prevention uses patient characteristics to filter a collection of preventive guidelines and to show only the guidelines that are relevant to that patient. A physician selects a patient's age, sex, and appropriate risk factors. Tapping recommendations shows a list of applicable reminders from the software's database.	Physicians documented all preventive measures performed or discussed during the patient visit in the usual way.
**Prytherch 2006 ** [**34**]	Crossover RCT; *Country*: UK; *Device*: PDA; *Media*: Application software	42 nurses	To compare the speed and accuracy of charting the weighted value attributed to each vital sign, and of calculating an EWS, using the traditional pen and paper method with that using the PDA. We also assessed nurses' preference for each system.	The hospital has developed a system for direct input of vital signs data into handheld PDAs, linked via Wi-Fi to a central computer. In the intervention arm EWS report was filled in and calculated using a PDA.	In the control arm the nurse would need to know or consult EWS weightings.
**Roy 2009 ** [**35**]	Cluster RCT; *Country*: France; *Device*: Other handheld computer; *Media*: Custom software	20 physicians in emergency departments looking at outpatients with clinically suspected pulmonary embolism.	To assess the effectiveness of a handheld clinical decision-support system to improve the diagnostic work-up of suspected pulmonary embolism among patients in the emergency department.	Physicians in the intervention group had a CDSS activated in their handheld devices during the intervention period. A physician who uses the program is first asked for the clinical variables necessary to generate a revised Geneva score that predicts the probability of pulmonary embolism. Duration: 7 mo	Physicians in the control groups used posters and pocket cards that showed validated diagnostic strategies
**Rudkin 2006 ** [**36**]	Crossover RCT; *Country*: USA; *Device*: PDA; *Media*: Application software	60 emergency medical residents or attendees a level 1 American College of surgeons-verified trauma centre or one of their patients.	To determine whether (1) patients accept EPs use of PDAs (2) EPs access PDAs or paper resources (either pocket or comprehensive textbooks) more frequently, (3) access time to electronic and pocket paper references differ, and (4) EPs with PDAs change patient diagnosis, drug therapy, or disposition more often than EPs with paper resources.	The intervention was a Handera 330 PDA that was preloaded with: pharmacopeia's, a general disease text, an infectious disease drug guide, and a medical calculator. Duration: 4 mo	In the control segment of the study residents carried text versions of the Tarascon Pharmacopeia and the Sanford Guide to Antimicrobial Therapy in their pockets. Text versions of Five Minute EM Consult and standard comprehensive EM texts were available.
**Schell 2006 ** [**37**]	Crossover RCT; *Country*: USA; *Device*: PDA	62 volunteers from fire and rescue and first responder organisations	To test the feasibility of automated handheld computer triage and compare it to written triage	8 disaster scenarios were created with table-2-captiona range of complexity. Participants in the intervention group used the PDA program, TriageDoc, table-2-caption which was developed to accommodate different triage methods from basic tag colour to RTS, TS, and elapsed time. From this basic input data, Glasgow Coma Score, RTS, and TS are calculated and the entry is time stamped ready for the next patient to be triaged.	Control participants manually documented the scenario.
**Skeate 2007 ** [**38**]	Parallel group RCT; *Country*: USA; *Device*: PDA; *Media*: Custom software	30 pathology residents (first through fifth year) or post-sophomore fellows.	To test if a PDA-based knowledgebase of surgical pathology report content recommendations improved report completeness.	The 15 experimental group and 13 control group residents were given microscope slides and corresponding reports with the final diagnosis section blanked-out, and were asked to complete the final diagnosis section during 3 study episodes (T0, T1, and T2). T0 and T2, neither group was allowed to use the knowledgebase; T1, experimental group was allowed to use the knowledgebase.	Pathology residents in the control arm, were provided a microscope, and unique surgical pathology “cases” each consisting of microscope slides and partially completed report templates, and were asked to complete the reports
**You 2009 ** [**39**]	Parallel group RCT; *Country*: Korea	49 junior medical students with no previous procedural experience. Mean age: Control = 26.1 y (SD 1.9), Intervention = 26.2 y (SD 1.7). Males: Control = 72%, Intervention = 75%	To determine if mobile VT could be used to facilitate an emergency method of instruction for the accurate performance of needle thoracocentesis.	All participants were given a 45-min lecture on the normal anatomy of the thorax, the pathophysiology and diagnosis of a pneumothorax, and needle thoracocentesis as treatment. The students were presented with a simulated scenario and a manikin involving a traumatic tension pneumothorax and were asked to perform needle thoracocentesis on the manikin. The intervention group performed the procedure under the guidance of VT, and could obtain standardized instructions from experienced emergency physicians on a real-time basis.	The control group performed the procedure without VT-aided instruction.

BP, blood pressure; CADM, computer assisted decision making; CDSS, clinical decision support system; EP, emergency physician; EWS, early warning score; ICU, intensive care unit; NSAID, non-steroidal anti-inflammatory drug; RCT, randomized controlled trial; RTS, revised trauma score; SD, standard deviation; TS, trauma score; VT, video telephony.

**Table 3:** Interventions applied for communication to or between health care providers.

Study	Study Design, Country, Device, Media	Participants	Aims	Intervention	Comparator
**Chandhanayingyong 2007 ** [**40**]	Diagnosis validation; *Country*: Thailand; *Device*: Mobile telephone; *Media*: MMS	Clinical staff with varying experience looked at 720 single, non- or minimally displaced fracture images.	To investigate the accuracy and usefulness of teleconsultation using the mobile phone MMS in emergency orthopaedic patients.	Digital X-ray images were sent via MMS to another mobile phone. The display size was 36–42 mm, magnification or adjustment of the image was not possible due to limitations of the mobile phone. Brief information regarding the history of the injury together with basic demographic data, including the age of each patient and data regarding important physical examination of each case was given. The assessors determined whether each case had a fracture or not, including the location of the fracture, and decided on the definitive treatment. Control = 91.2% male; intervention = 96.25% male.	Both clinical and radiographic follow-up data was used as a gold standard.
**Eze 2005 ** [**41**]	Prospective case controlled series; *Country*: UK; *Device*: Mobile telephone; *Media*: Picture	14 randomly selected patients who underwent emergency ear, nose and throat procedures over a 1-mo period.	To determine the accuracy of assessment of common ENT emergency radiological investigations using mobile phone digital images.	CT scans and X-ray images taken from and transmitted via a mobile phone via MMS to another phone of the same make and model. Received images were shown sequentially to senior members of the otolaryngology department, including six consultants and five specialist registrars. A senior doctor off site in another hospital and the resident doctor on-site gave a brief history and transmitted the selected images via the mobile phone.	Usual care. The same X-ray films were examined using an X-ray box.
**Gandsas 2004 ** [**42**]	Crossover RCT; *Country*: USA; *Device*: Other handheld computer; *Media*: MP4/video	46 surgical residents who had completed an endoscopy rotation.	To determine whether the images transmitted wirelessly to a handheld computer are adequate to allow a physician to accurately identify the anatomy and thus allow a surgeon to potentially telementor during an on-going procedure on the basis of these images.	Two previously recorded endoscopic procedures were used. Each participant was first assigned to a viewing device, standard screen or handheld computer, and to a video, tape 1, or tape 2. Each participant was given a ten question quiz to be completed while viewing the corresponding Tape. Both videos contained ten anatomical landmarks marked by a black arrow and a number (1–10), which corresponded to a 5-option MCQ asking for the name of the highlighted structure. Participants were allowed to pause the tape while answering each question.	Standard screen view of the video was used as the control in this intervention.
**Hsieh 2005 ** [**43**]	Non-randomized parallel group trial; *Country*: Taiwan; *Device*: Mobile telephone; *Media*: MMS	120 digital complete amputation patients.	To evaluate the feasibility of clinical application of the camera-phone for remote diagnosis and recommendations about replantation potential in those cases presenting with complete digital amputation.	35 patients with 60 digital complete amputations were admitted to the ER. The picture of the amputated part and stump of the injured digit(s) was transmitted to another camera-phone held by the remote consultant surgeon, to be reviewed on the display screen. Next, a brief medical and trauma history of each patient was relayed by mobile phone, with further discussion to clarify the condition. Duration: 10 mo	The consultant surgeon visited and reviewed all of these patients and completed the same standard wound questionnaire after on-site inspection.
**Ortega 2009 ** [**44**]	Parallel group RCT; *Country*: USA; *Device*: Mobile telephone; *Media*: Voice	Selected orthopaedic residents, attendees, and orthopaedic floor nurses.	To compare floor nurse and intraoperative surgeon communication (nurse to surgeon, and surgeon to nurse).	Cellular communication using a blue-tooth wireless earpiece was used instead of the usual, indirect form of pager communication used between floor nurses and surgeons during surgery to assess for improved communication times.	Usual procedure of contacting the surgeon during surgery: the floor nurse contacted the operating room by pager; this was picked up by circulating nurse and communication proceeded between floor nurse, circulating nurse, and surgeon whilst the surgeon was operating.
**Pettis 1999 ** [**45**]	Non-randomised cross-over study; *Country*: USA; *Device*: Other handheld computer; *Media*: Custom software	30 cardiologists	To compare the intra-observer agreement of physicians' interpretations of 12-lead ECGs on traditional thermal paper to interpretations made from the LCD screens of hand-held computers.	The study participants were information including an answer sheet of 39 different ECG diagnoses and a HP Palmtop computer containing 20 sample ECGs. The participants were instructed to select a diagnosis from the answer sheet and given no restrictions as to the number of times that a certain answer could be used. The participants indicated their diagnoses for all 20 sample ECGs and returned their answer sheets.	1 mo after receipt of the LCD-displayed ECG interpretations, the same 20 ECGs were sent in printed hard copy form to each participant. The participants entered their diagnoses on the answer sheet and then returned both ECGs and answer sheet to the Lab.
**Yaghmai 2003 ** [**46**]	Non-randomized parallel group trial; *Country*: USA; *Device*: PDA; *Media*: MMS	42 scans that had been taken to exclude inter-cranial haemorrhage in patients following acute trauma.	To assess the feasibility of using a PDA as a medium for the interpretation of cranial CT scans of trauma patients.	21 complete CT scans were saved by a radiologist onto the hard drive of a computer; all had previously been obtained to exclude inter-cranial haemorrhage following acute trauma. The studies on the PDA were separately evaluated by a radiologist and a neurosurgeon, assessed for image quality as well as intracranial haemorrhage.	Cranial CT scans had been interpreted by a board-certified radiologist prior to the study

CT, computerized tomography; ENT, ear nose and throat; ER, emergency room; LCD, liquid crystal display; MCQ, multiple choice questionnaire; RCT, randomized controlled trial.

#### Communication between health care services and health care consumers

The ten trials recruited 4,473 participants with sample sizes ranging from 31 to 1,859 participants. Seven were randomised controlled trials with parallel groups and three were non-randomised parallel group trials. Of the ten trials of health services support, eight were trials of short messaging service (SMS, text message)-based appointment reminders [47]–[54] (Table 4) and two were trials of SMS-based patient notification of results [55],[56] (Table 5). Four appointment reminder trials were conducted in high-income countries and three were conducted in middle-income countries. Both the trials of patient notification of test results were conducted in high-income countries.

**Table 4:** Interventions applied for health services support: appointment reminders.

Study	Study Design, Country, Device, Media	Participants	Aims	Intervention	Comparator
**Bos 2005 ** [**47**]	Non-randomised parallel group trial; *Country*: Holland; *Device*: Mobile telephone; *Media*: SMS	301 patients with appointments an orthodontic clinic.	The aim of this study was to retest the hypotheses of Reekie and Devlin (1998) [70]. It was hypothesized that a reminder would reduce the failed attendance rate.	Patients received a reminder text (intervention 2) or telephone call (intervention 1), 1 d before the appointment. Duration: 3 wk.	No reminder, reminder phone call, and a reminder letter (mail).
**Chen 2008 ** [**48**]	Parallel group RCT; *Country*: China; *Device*: Mobile telephone; *Media*: SMS	1,859 people with scheduled appointments at the health promotion centre of Sir Run Run Shaw Hospital, Zhejiang that fell during the study period. Mean age: control (no reminders) = 51.14 y (range = 39.22–63.06), (telephone reminder) = 50.52 y (range = 38.99–62.05); intervention = 50.01 y (range = 39.02–61.0).	To compare the efficacy of a SMS text messaging and phone reminder to improve attendance rates at a health promotion centre.	A reminder was sent to both SMS and telephone groups 72 h prior to the appointment. The reminder was similar in content including participant's name and appointment details. Duration: 2 mo.	No reminder.
**Da Costa 2010 ** [**49**]	Non-randomised parallel group trial; *Country*: Brazil; *Device*: Mobile telephone; *Media*: SMS	Patients attending outpatient clinics that were KATU clients and used their SMS appointment reminder system (29,014 appointments)	To study the impact of appointment reminders sent as SMS text messages to patients' cell phones on nonattendance rates.	Data on SMS appointment reminders sent and also about attendance and nonattendance at scheduled appointments were obtained. Duration: 11 mo.	No reminder.
**Fairhurst 2008 ** [**50**]	Parallel group RCT; *Country*: Scotland; *Device*: Mobile telephone; *Media*: SMS	418 patients who failed to attend two or more routine doctor or nurse appointments in the preceding 12 mo and made an appointment during the study time. Mean age: control = 33.1 y (SD = 9.8); intervention = 33.1 y (SD = 10).	To evaluate the effectiveness of texting appointment reminders to patients who persistently fail to attend appointments.	The intervention comprised a text message reminder of the appointment sent between 8:00 and 9:00 on the morning preceding afternoon appointments and between 16:00 and 17:00 on the afternoon preceding morning appointments. Duration: 6–7 mo	No reminder.
**Fung 2009 ** [**51**]	Parallel group RCT; *Country*: USA; *Device*: Mobile telephone; *Media*: SMS	31 repeat blood donors.	To study the effectiveness of text message reminders versus usual phone reminders on donor show rates with scheduled donation appointments.	Donors received a text message reminder of a scheduled appointment with the blood donation clinic. Duration: 7 d	Usual phone reminders.
**Leong 2006 ** [**52**]	Parallel group RCT; *Country*: Malaysia; *Device*: Mobile telephone; *Media*: SMS, Voice	993 patients with time-appropriate appointments and who had (or their caregivers) a mobile phone with text messaging function. Mean age: Control = 37.8 y; intervention (mobile phone call) = 38.4 y (SMS reminder) = 38.4.	To determine the effectiveness of a text messaging reminder in improving attendance in primary care.	In both intervention groups, a reminder was sent using a mobile phone 24 to 48 h prior to the appointment. The text messaging and mobile phone messages consisted of patient's name and appointment time. The mobile phone conversation was similar to the text messaging reminder message and no clinical or laboratory information was included. Duration: 6 mo.	No reminder.
**Liew 2009 ** [**53**]	Parallel group RCT; *Country*: Malaysia; *Device*: Mobile telephone; *Media*: SMS	931 participants registered with the clinics for at least 6 mo who had at least one chronic disease, a return appointment between 1 and 6 mo and ownership of a mobile phone. Mean age: control (no reminder) = 60.77 y, (telephone call) = 57.73 y; intervention (text reminder) = 58.19 y.	To determine if text messaging would be effective in reducing non-attendance in patients on long-term follow-up, compared with telephone reminders and no reminder.	Reminders were sent to the participants 24–48 h before the scheduled appointment. To avoid caller bias during telephone conversations, a research assistant was trained to deliver the same telephone message as in the telephone reminder.	No reminder or a telephone call delivered in a standard way by a trained research assistant.
**Milne 2006 ** [**54**]	Parallel group RCT; *Country*: UK; *Device*: Mobile telephone; *Media*: SMS	16,400 patients with an appointment booked in Yorkhill Hospital in either Aug or Sept 2004.	To assess DNA rates for those receiving SMS reminders and those who didn't receive SMS reminders.	Patients are contacted one working day prior to the appointment date and the same message is sent to all patients, being “this is a reminder of an appointment at Yorkhill on DATE and TIME. For enquiries or to cancel please call XXX.” Duration: 2 mo.	No reminder.

DNA, did not attend; RCT, randomized controlled trial; SD, standard deviation.

**Table 5:** Interventions applied for health services support: test result notification.

Study	Study Design, Country, Device, Media	Participants	Aims	Intervention	Comparator
**Cheng 2008 ** [**55**]	Parallel group RCT; *Country*: Taiwan; *Device*: Mobile telephone; *Media*: SMS	Pregnant women attending the Chang Gung Memorial Hospital, Taiwan, who could speak Chinese and who agreed to undergo Down Syndrome screening.	To study the effect of fast reporting by mobile phone SMS on anxiety levels in women undergoing prenatal biochemical screening for Down Syndrome.	Pregnant women were given appointments for regular clinical follow-up after serum testing for Down Syndrome. If the serum screening results were negative, group A were sent a pre-clinic SMS.	Group B were not offered fast reporting
**Menon-Johansson 2006 ** [**56**]	Concurrent Cohort Study; *Country*: UK; *Device*: Mobile telephone; *Media*: SMS	78 patients with a diagnosis of untreated genital CT infection. Mean age: Control = 27.2 y (SD 8.6), 95.2% female; intervention = 24.8 y (SD 3.9), 96.4% female.	To assess the effectiveness of a text message result service within an inner London sexual health clinic.	Patients with a diagnosis of untreated genital CT who were sent a text message and compared to patients with untreated CT recalled by standard methods. Texts were one of the following 3: “all of your results are negative,” “please ring the clinic,” “please come back to the clinic.”	Usual care: patients were asked to return to the clinic, or phone the clinic for results.

RCT, randomized controlled trial.

### Interventions

The interventions are described according to the authors' descriptions in Tables 1–5. Below we describe the interventions according to the functions employed (SMS messaging, photos, video, application software, telephone, and multimedia messages [MMS]) and devices employed (e.g., PDA, mobile phone, hand-held computer, and portable media player).

#### Health care provider support

For the medical education interventions, six used application software delivered via personal digital assistants [15]–[20]. One trial employed a MP4/video technology using a portable media player [21].

For interventions supporting clinical diagnosis and management, 14 trials used customised application software (12 on personal digital assistants, one on a tablet PC, one on a handheld computer). Four trials used photographs and video capabilities using mobile phones.

For interventions using mobile technologies to communicate between health care providers for clinical/patient management, three trials [40],[43],[46] relied on the use of MMS for sending images by mobile phone, and one trial used the telephone function of the mobile phone [44]. One trial used MMS on a PDA. One trial made use of MP4/video technology and the other made use of installed customised software, using hand-held computers.

#### Communication between health services and consumers

All the interventions used SMS messages delivered by mobile phone. One appointment reminder trial also used voice messages. Details of the control groups are provided in Tables 1–5. In the medical education trials, the control groups were medical education delivered via a range of standard traditional media. For the diagnosis and clinical management trials and health, verbal, and data communication trials, the control groups were standard care or standard methods. In six appointment reminder trials, the control group was no reminder; in two reminder trials, the comparison group was a telephone reminder; and in one the comparison group was a letter.

### Outcomes

#### Health care provider support

The trials reported between one and 15 outcomes. Nineteen of the 28 trials provided sufficient data to calculate effect estimates.

For primary outcomes, there were no objective measures of health or health service delivery reported.

In terms of secondary outcomes, for medical education interventions, one trial reported two outcomes regarding documentation of health care problems [19] and four trials reported nine knowledge outcomes [16],[18],[19],[21]. For clinical diagnosis and management interventions, seven trials [28],[30],[33]–[37] reported 25 outcomes relating to appropriate management (3 outcomes), testing (3) [28], referrals (1) [28], screening (4) [33], diagnosis (2) [34],[35], treatment (2) [35],[36], and triage (10) [37]. Six trials [26],[34],[36],[38],[39] reported 17 medical process outcomes: perceived difficulty in performing a task (1 outcome) [39], use of tool (1) [36], errors in report (2), errors in score calculation (2) [34], completeness of reports (2) [38], time to complete a report (2) [38], time to record vital signs (1) [34], time to diagnosis (3) [26], and time to treatment (3) [26]. For interventions using mobile technologies to communicate between health care providers for clinical/patient management outcomes, six trials [40],[42]–[44],[46],[57] reported 19 outcomes relating to the quality of nurse surgeon communication (6 outcomes) [44], correct clinical assessment or diagnosis (4) [40],[43],[46], test score (1) [42] and electrocardiogram (ECG) transmission (8) [57], feasibility of delivery (1), time taken (4), and quality (3) [57]).

#### Communication between health services and consumers

The trials reported between one and three outcomes. Nine of the ten trials provided sufficient data to calculate effect estimates.

For primary outcomes, eight trials reported appointment attendance as an outcome [47]–[54] and two trials reported cancelled appointments as an outcome [47].

In terms of secondary outcomes, for patient notification of test results, outcomes were the following: time to diagnosis (1 outcome), time from first contact to treatment (1) and time from test to treatment (1), and anxiety scores (2) [55],[56].

### Study Quality

#### Health care provider support

**Figure 2:**
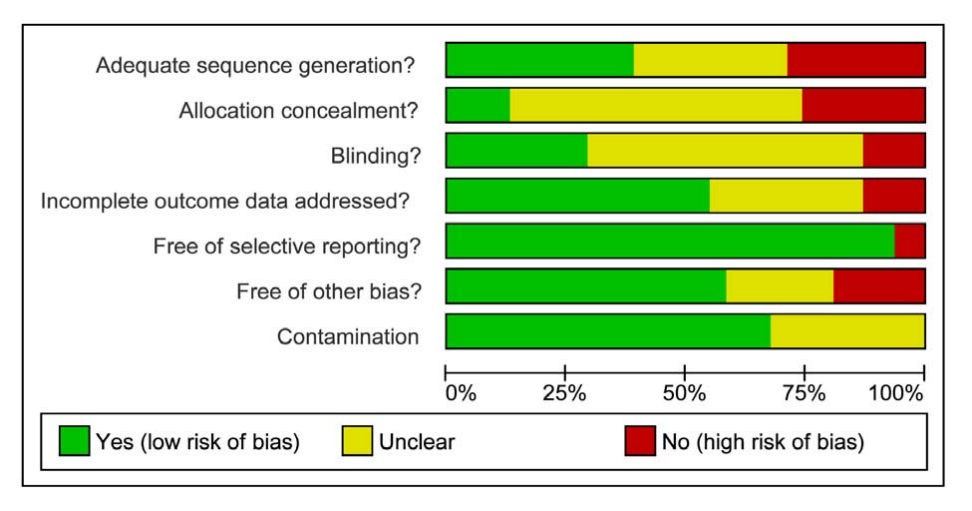
Cochrane summary risk of bias for trials of health care provider support interventions (n = 32).

The assessment of **s**tudy quality is reported in Table 6 and the Cochrane risk of bias summary is reported in Figure 2.

**Table 6:** Cochrane risk of bias summary for health care provider support trials.

Trial	Sequence Generation	Allocation Concealment	Blinding	Incomplete Outcome Data	Selective Outcome Reporting Bias	Contamination	Other Bias
**Medical education**							
Farrell 2008 [15]	U	U	L	H	L	L	U
Mcleod 2009 [19]	L	U	L	L	L	L	H
Mcleod 2006 [18]	U	U	U	U	H	U	U
Goldsworthy 2006 [16]	L	L	L	H	L	L	L
Leung 2003 [17]	L	L	U	L	L	L	L
Strayer 2010 [20]	U	U	L	U	H	L	L
Tempelhof 2009 [21]	L	L	L	L	L	U	U
**Clinical diagnosis and management support**							
Berner 2006 [22]	L	U	L	L	L	L	L
Bürkle 2008 [24]	L	U	H	U	L	L	L
Coopmans 2008 [26]	L	U	U	L	L	L	H
Greenfield 2007 [27]	H	H	U	L	L	U	H
Greiver 2005 [28]	L	U	U	H	L	L	L
Jayaraman 2008 [29]	L	U	U	L	L	L	U
Lee 2009 [30]	U	U	U	U	L	U	L
Mclaughlin 2010 [71]	U	U	U	U	L	L	U
Momtahan 2007 [32]	H	H	U	U	L	U	H
Price 2005 [33]	L	U	H	L	L	L	L
Prytherch 2006 [34]	U	U	U	L	L	L	L
Roy 2009 [35]	L	L	L	U	L	L	L
Rudkin 2006 [36]	H	H	U	L	L	U	L
Schell 2006 [37]	U	U	U	L	L	L	L
Skeate 2007 [38]	U	U	U	H	L	L	L
You 2009 [39]	L	U	U	L	L	L	L
**Communication between health care providers for clinical/patient management**							
Blaivas 2005 [23]	H	H	H	L	L	L	H
Chandhanayingyong 2007 [40]	H	H	L	U	L	U	L
Gandsas 2004 [42]	U	U	U	U	L	U	L
Hsieh 2005 [43]	H	H	H	L	L	L	L
Mclaughlin 2010 [31]	U	U	U	U	L	L	U
Ortega 2009 [44]	U	U	U	U	L	L	H
Pettis 1999 [45]	H	H	U	L	L	U	L
Vaisanen 2003 [57]	H	U	U	L	L	L	U
Yaghmai 2003 [46]	H	H	L	L	L	U	U

L, low; H, high; U, unclear.

#### Communication between health services and consumers

**Figure 3:**
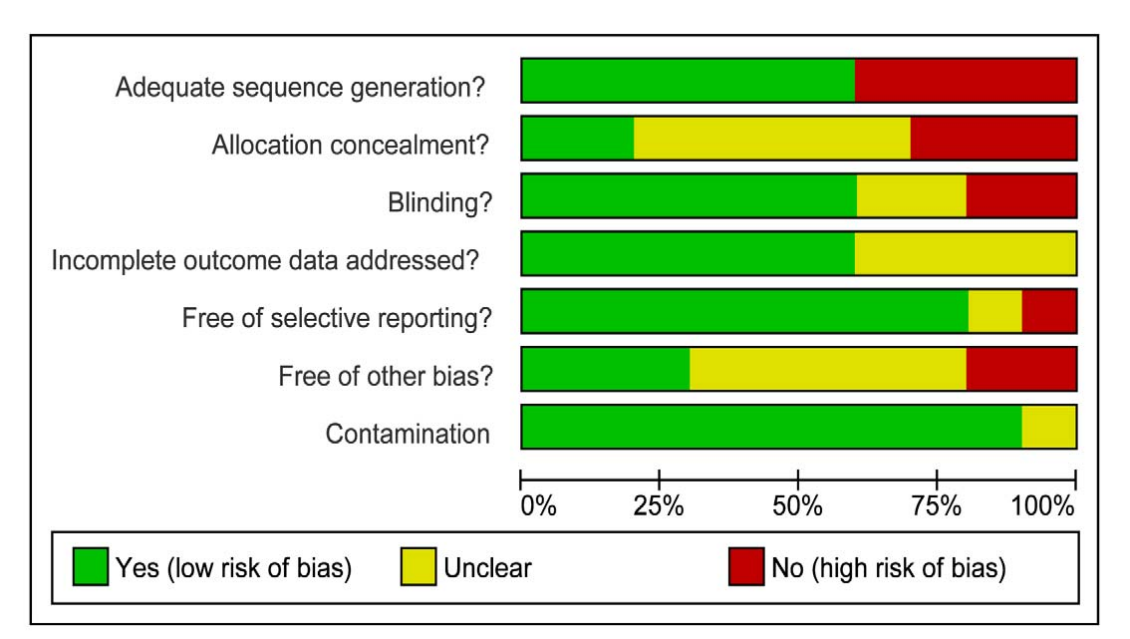
Cochrane summary risk of bias for trials of health services support (n = 10).

The assessment of **s**tudy quality is reported in Table 7 and the Cochrane risk of bias summary is reported in Figure 3.

**Table 7:** Cochrane risk of bias summary for health service support trials.

Trial	Sequence Generation	Allocation Concealment	Blinding	Incomplete Outcome Data	Selective Outcome Reporting Bias	Contamination	Other Bias
**Appointment reminders**							
Bos 2005 [47]	H	U	L	L	H	L	L
Chen 2008 [48]	L	U	L	L	L	L	U
Da Costa 2010 [49]	H	H	H	U	L	L	U
Fairhurst 2008 [50]	L	L	L	L	L	L	H
Fung 2009 [51]	H	H	H	U	U	U	U
Leong 2006 [52]	L	U	L	L	L	L	U
Liew 2009 [53]	L	L	L	U	L	L	U
Milne 2009 [54]	L	U	L	L	L	L	L
**Test result**							
Cheng 2008 [55]	L	U	U	U	L	L	L
Menon-Johansson 2006 [56]	H	H	U	L	L	L	H

L, low; H, high; U, unclear.

None of the trials were at low risk of bias for all quality criteria. There was no evidence of publication bias on visual and statistical examination of funnel plots.

### Effects

We report the effect estimates for primary outcomes and a summary of the effect estimates for secondary outcomes (see Tables 8–12 for the secondary outcome effect estimates).

**Table 8:** Effect estimates for trials of medical education interventions.

Trial	Intervention	Outcome	RR	MD	LCI	UCI
**Secondary Outcomes**						
Mcleod 2009 [19]	PDA assessment tool versus PDA without tool	Tabulation of geriatric functional issues on dismissal summaries	0.98	—	0.62	1.54
Mcleod 2009	PDA assessment tool versus no device	Tabulation of geriatric functional issues on dismissal summaries	0.96	—	0.65	1.43
Tempelhof 2009 [21]	Conference talks on MP3/MP4 versus attendance at conference	Conference 1 MCQ correct	1.07	—	0.39	2.92
Tempelhof 2009	Conference talks on MP3/MP4 versus attendance at conference	Conference 2 MCQ correct	1.07	—	0.66	1.74
Tempelhof 2009	Conference talks on MP3/MP4 versus attendance at conference	Conference 3 MCQ correct	1.19	—	0.70	2.02
Tempelhof 2009	Conference talks on MP3/MP4 versus attendance at conference	Conference 4 MCQ correct	1.07	—	0.66	1.74
Tempelhof 2009	Conference talks on MP3/MP4 versus attendance at conference	Conference 5 MCQ correct	0.95	—	0.52	1.76
Tempelhof 2009	Conference talks on MP3/MP4 versus attendance at conference	Overall MCQ correct	1.07	—	0.61	1.89
Goldsworthy 2006 [16]	PDA loaded with clinical reference material versus manual access to material	Test score (medical education)	—	3.14	0.73	5.56
Mcleod 2009	PDA assessment tool versus no device	Test score (no device versus PDA)	—	0.23	−0.65	1.11
Mcleod 2009	PDA assessment tool versus no device	Test score (PDA without tool versus PDA)	—	0.05	−0.97	1.07

LCI, lower confidence interval; MCQ, multiple choice questionnaire; MD, mean deviation; UCI, upper confidence interval.

**Table 9:** Effect estimates for trials of clinical diagnosis and management support: appropriate management outcomes (testing, referrals, screening, diagnosis, treatment, or triage).

Trial	Intervention	Outcome	RR	MD	LCI	UCI
Griever 2005 [28]	PDA software diagnosis aid versus no device	Appropriateness of referral for cardiac stress testing	1.61	—	0.81	3.18
Griever 2005	PDA software diagnosis aid versus no device	Appropriateness of referral for nuclear cardiology testing after cardiac stress testing.	1.45	—	0.88	2.38
Griever 2005	PDA software diagnosis aid versus no device	Proportion referred to cardiologist	0.96	—	0.52	1.79
Griever 2005	PDA software diagnosis aid versus no device	Proportion of participants referred for cardiac stress tests	1.57	—	1.04	2.36
Lee 2009 [30]	CDSS on PDA - obesity-related diagnoses versus paper resource	Encounters with missed obesity related diagnosis	0.37	—	0.30	0.45
Lee 2009	CDSS on PDA - obesity-related diagnoses versus paper resource	Encounters with obesity related diagnosis	12.49	—	6.34	24.62
Price 2005 [33]	CDSS on PDA versus no device	Proportion of patients eligible for hypertension screen that received it	0.98	—	0.87	1.09
Price 2005	CDSS on PDA versus no device	Proportion of patients eligible for lipid disorder screen that received it	1.50	—	1.16	1.96
Price 2005	CDSS on PDA versus no device	Proportion of patients eligible for pap test that received it	1.15	—	1.01	1.30
Price 2005	CDSS on PDA versus no device	Proportion of patients eligible for prophylactic use of acetylsalicylic acid that received it	2.60	—	1.58	4.27
Price 2005	CDSS on PDA versus no device	Proportion of patients eligible for colorectal cancer screen that received it	1.81	—	1.12	2.92
Prytherch 2006 [34]	Clinical chart on PDA versus no device	Incorrect clinical actions (based on recording of vital signs and calculation of early warning scores)	0.14	—	0.01	2.61
Roy 2009 [35]	CDSS on handheld computer versus no device	Appropriate diagnostic work-up	2.50	—	0.63	10.00
Rudkin 2006 [36]	PDA loaded with clinical guides versus no device	Change in diagnosis, treatment of disposition management (excluding drugs)	2.00	—	0.19	20.90
Rudkin 2006	PDA loaded with clinical guides versus no device	Change in drug (interaction, dose, cost, indication) choice	2.00	—	0.55	7.27
Schell 2006 [37]	Automated versus manual computer triage	Correct identification of critical patients using triage score: Fire	—	0.60	−1.07	2.27
Schell 2006	Automated versus manual computer triage	Correct identification of critical patients using triage score: MVA	—	−0.70	−2.10	0.70
Schell 2006	Automated versus manual computer triage	Correct identification of critical patients using triage score: Practice	—	4.30	2.51	6.09
Schell 2006	Automated versus manual computer triage	Correct identification of critical patients using triage score: Total score	—	5.30	−0.27	10.87
Schell 2006	Automated versus manual computer triage	Correct identification of critical patients using triage score: mass casualty index score	—	1.10	−2.08	4.28
Schell 2006	Automated versus manual computer triage	Triage time: Fire	—	0.60	−0.08	1.28
Schell 2006	Automated versus manual computer triage	Triage time: MVA	—	−0.80	−1.53	−0.07
Schell 2006	Automated versus manual computer triage	Triage time: Practice	—	−1.00	−1.96	−0.04
Schell 2006	Automated versus manual computer triage	Triage time: Total score	—	−3.20	−5.27	−1.13
Schell 2006	Automated versus manual computer triage	Triage time: mass casualty index	—	−1.90	−3.00	−0.80

LCI, lower confidence interval; MVA, motor vehicle accident; UCI, upper confidence interval.

**Table 10:** Effect estimates for trials of clinical diagnosis and management support: medical process outcomes.

Clinical Trial	Intervention	Outcome	RR	MD	LCI	UCI
Prytherch 2006 [34]	Clinical chart on PDA versus no device	Error in calculation of early warning score (incorrect data)	0.20	—	0.03	1.57
Prytherch 2006	Clinical chart on PDA versus no device	Error in calculation of early warning score (missing data)	3.00	—	0.13	69.70
Prytherch 2006	Clinical chart on PDA versus no device	Errors in report (incorrect data)	0.33	—	0.01	7.74
Prytherch 2006	Clinical chart on PDA versus no device	Errors in report (missing data)	0.33	—	0.01	7.74
Rudkin 2006 [36]	PDA loaded with clinical guides versus no device	Percent use of clinical guide - emergency medicine	1.04	—	0.89	1.21
Skeate 2007 [38]	PDA knowledgebase versus no device	Diagnosis reports felt to be complete, but were not at 48 h follow-up test (T2)	0.58	—	0.35	0.95
Skeate 2007	PDA knowledgebase versus no device	Diagnosis reports felt to be complete, but were not at 48 h follow-up test (T1)	0.69	—	0.40	1.21
Coopmans 2008 [26]	CDSS on PDA versus no device	Case 1: Mean time to correct diagnosis	—	7.85	2.14	13.56
Coopmans 2008	CDSS on PDA versus no device	Case 1: Mean time to recognize abnormal event	—	0.65	−0.01	1.31
Coopmans 2008	CDSS on PDA versus no device	Case 1: Mean time to definitive treatment	—	8.00	1.14	14.86
Coopmans 2008	CDSS on PDA versus no device	Case 2: First indicates correct diagnosis	—	−16.58	−27.66	−5.50
Coopmans 2008	CDSS on PDA versus no device	Case 2: Mean time to definitive treatment.	—	−17.31	−21.23	−13.39
Prytherch 2006	Clinical chart on PDA versus no device	Completion time for report of vital signs including calculation of early warning score.	—	−24.60	−42.74	−6.46
Skeate 2007	PDA knowledgebase versus no device	Time spent completing a diagnosis report	—	185.50	−626.72	997.72
Skeate 2007	PDA knowledgebase versus no device	Time spent completing a diagnosis report at 48 h follow-up test	—	6.50	−721.75	734.75
You 2009 [39]	Video telephony for medical procedure versus no device	Difficulty in performing needle thoracocentesis	—	−2.30	−3.15	−1.45
You 2009	Video telephony for medical procedure versus no device	Time to success: needle thoracocentesis performance	18.20	—	5.63	5.63

LCI, lower confidence interval; MD, mean deviation; UCI, upper confidence interval.

**Table 11:** Effect estimates for health service support trials.

Trial	Intervention	Outcome	RR	MD	LCI	UCI
**Appointment reminder trials: primary outcomes**						
Bos 2005 [47]	SMS versus mail reminder	Cancelled appointments	2.67	—	0.92	7.71
Bos 2005	SMS reminder versus phone call	Cancelled appointments	2.31	—	0.90	5.95
Leong 2006 [52]	Mobile phone call versus no reminder	Appointment attendance	1.24	—	1.07	1.43
**Test result notification trials: secondary outcomes**						
Menon-Johansson 2006 [56]	SMS notification of test results versus no SMS	Mean time to communication of diagnosis	—	−5	−6.94	−2.26
Menon-Johansson 2006	SMS notification of test results versus no SMS	Mean time from first contact to treatment	—	0	−0.44	0.44
Menon-Johansson 2006	SMS notification of test results versus no SMS	Mean time from test to treatment	—	−6	−7.15	−4.85

LCI, lower confidence interval; MD, mean deviation; UCI, upper confidence interval.

**Table 12:** Effect estimates for trials of interventions to facilitate communication between health care professionals for clinical/patient management.

Trial	Intervention	Outcome	RR	MD	LCI	UCI
Chandhanayingyong 2007 [40]	Photo of X-ray on mobile phone versus gold standard	Fracture detection	0.79	—	0.76	0.82
Hsieh 2005 [43]	Photo of amputation injury on mobile phone versus gold standard	Correct assessment of potential to perform re-implantation	0.90	—	0.82	0.99
Hsieh 2005	Photo of amputation injury on mobile phone versus gold standard	Recognition of skin ecchymoses	0.79	—	0.69	0.90
Ortega 2009 [44]	Mobile call between nurse and surgeon versus usual practice	Nurse - Surgeon: Call refusal or delay	0.14	—	0.01	2.65
Ortega 2009	Mobile call between nurse and surgeon versus usual practice	Nurse - Surgeon: intra-operative case interruption.	0.05	—	0.00	0.87
Ortega 2009	Mobile call between nurse and surgeon versus usual practice	Nurse - Surgeon: Communication difficulties	0.14	—	0.01	2.65
Ortega 2009	Mobile call between nurse and surgeon versus usual practice	Nurse - Surgeon: Intra-operative noise interference	0.20	—	0.01	4.00
Ortega 2009	Mobile call between nurse and surgeon versus usual practice	Nurse - Surgeon: response rate	1.42	—	1.12	1.80
Ortega 2009	Mobile call between surgeon and nurse versus usual practice	Surgeon - Nurse: response rate	1.03	—	0.94	1.13
Vaisanen 2003 [57]	Fax transmitted via mobile phone usual procedure	Transmission times: transmission from fax via satellite	0.17	—	0.04	0.30
Vaisanen 2003	Fax transmitted via mobile phone usual procedure	Transmission times: transmission from table fax.	0.02	—	−0.15	0.19
Vaisanen 2003	Fax transmitted via mobile phone usual procedure	Transmission times: transmission from monitor defibrillator.	−0.02	—	−0.27	0.23
Vaisanen 2003	Fax transmitted via mobile phone usual procedure	Transmission times: transmission from mobile fax and phone.	1.20	—	−0.36	2.76
Vaisanen 2003	Fax transmitted via mobile phone usual procedure	Quality of transmitted ECG: transmission from fax via satellite	−0.10	—	−0.36	0.16
Vaisanen 2003	Fax transmitted via mobile phone usual procedure	Quality of transmitted ECG: transmission from table fax.	−0.30	—	−0.73	0.13
Vaisanen 2003	Fax transmitted via mobile phone usual procedure	Quality of transmitted ECG: transmission from mobile fax and phone.	−0.10	—	−0.53	0.33
Vaisanen 2003	Fax transmitted via mobile phone	Proportion of failed attempts during ECG transmission	1.00	—	0.07	14.79
Yaghmai 2003 [46]	Photo of CT scan on PDA versus gold standard	Diagnosis: percentage positive	0.91	—	0.77	1.07
Gandsas 2004 [42]	Recording of surgery on handheld computer versus standard screen	Score: test on video of two standard surgical procedures	—	−3.4	−10.3	3.5

LCI, lower confidence interval; MD, mean deviation; UCI, upper confidence interval.

### Health care provider support

No studies reported our primary outcomes.

The following secondary outcomes were reported.

*Medical education interventions:* Of the nine knowledge outcomes reported, eight showed no statistically significant effects and one showed a statistically significant increase in knowledge (Table 8). There were no statistically significant effects on the two reported outcomes regarding documentation.

*Clinical diagnosis and management support interventions:* Seven trials [28],[30],[33]–[37] using application software to deliver support reported 25 outcomes relating to appropriate management, testing, referrals screening, diagnosis, treatment, and triage; of these, 19 outcomes showed benefits of which 11 were statistically significant (Table 9). The other six outcomes showed no clinically important or statistically significant direction of effect (Table 9). For medical process measures (time for procedures, completeness of or errors in data/reports, perceived difficulty of procedures, diagnostic confidence) five trials [26],[34],[36],[38],[39] reported 17 outcomes; of these, five showed statistically significant benefits (Table 10). Six outcomes showed negative effects in increasing time for processes or errors in data, of which three were statistically significant. One outcome had no clear direction of effect.

*Interventions to facilitate verbal or data communication between health care providers:* The effect estimates are provided in Table 6. One trial [44] using a mobile phone to facilitate communication between nurses and surgeons reported six outcomes; one showed statistically significant benefit. Two trials [40],[43] using photos transmitted via mobile phones reported three outcomes showing negative effects of the interventions, with statistically significant reductions in fracture detection when compared to standard radiographic pictures, reductions in correct assessment of potential to perform re-implantation, and correct recognition of skin ecchymoses when compared to a gold standard assessment by a specialist evaluating ecchymoses in person. One trial [42] reported a nonsignificant reduction in the ability of doctors to interpret endoscopy videos when viewed on a hand-held computer compared to a standard monitor. One trial [57] compared an ECG transmitted via mobile phone to an ECG transmitted by fax and reported statistically significant reductions for one of three outcomes regarding ECG quality. The authors report there were no effects of this difference in quality on ECG interpretation but do not provided data on this. Of four reported outcomes regarding the time taken to transmit the ECG, none were statistically significant.

#### Communication between health services and consumers

**Figure 4:**
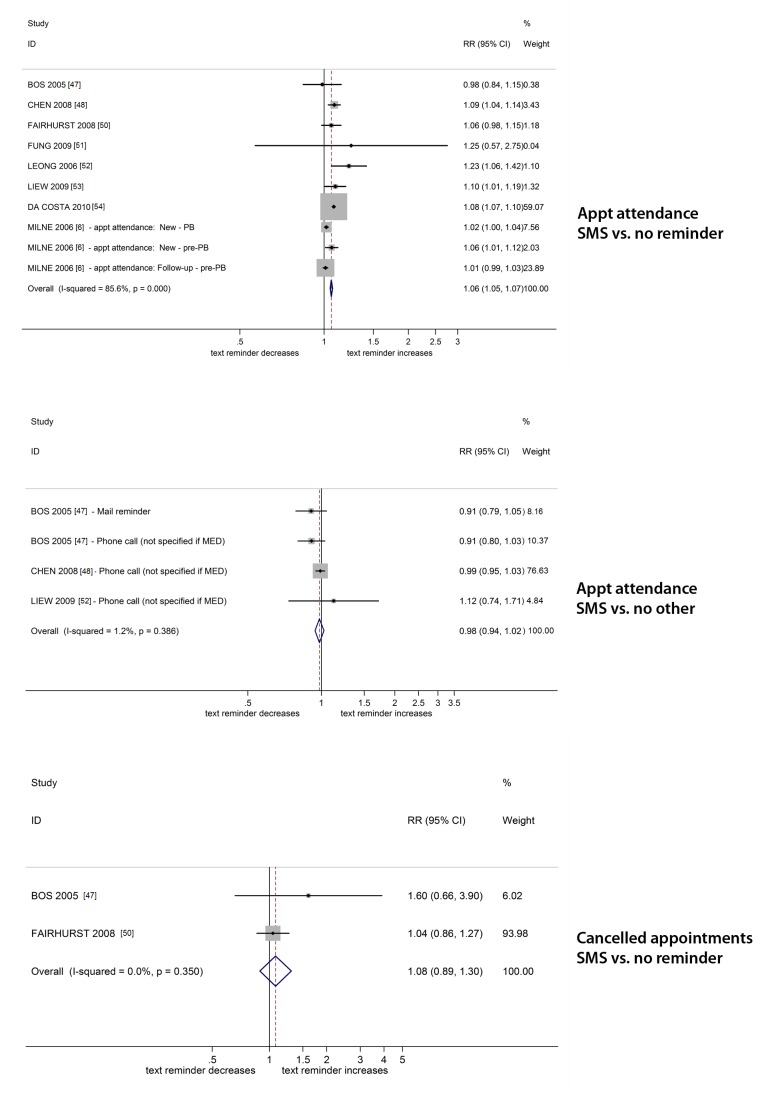
Forest plots of the effect of SMS reminders on appointments.

Primary outcomes were reported in eight trials [47]–[54] that evaluated the effect of attendance reminders using SMS reminders versus no reminder and showed a statistically significant increase in attendance (pooled relative risk [RR] 1.06 [95% CI 1.05–1.07], *I* ^2^ squared 86%). The pooled effect for trials evaluating the effect of attendance reminder using text message against reminders that used other modes, such as postal reminder and phone calls, showed no significant change (RR 0.98; 95% CI 0.94–1.02, *I* ^2^ = 1.2%). Two trials [47],[50] that evaluated the effects on cancellations of texting appointment reminders to patients who persistently fail to attend appointments showed no statistically significant change (pooled RR of 1.08; 95% CI 0.89–1.30, *I* ^2^ = 0%) (Figure 4). Another trial [47] reported the effects on appointment cancellation of mobile phone reminders compared to postal mail (RR 2.67; 95% CI 0.92–7.71) and phone call reminders (RR 2.31; 95% CI 0.91–5.95) (Table 11); both showed increases that were not statistically significant. One trial [52] evaluated the effect of appointment reminder by mobile phone call compared with a control group that received no reminder and showed a statistically significant increase in attendance (RR 1.24; 95% CI 1.07–1.43) (Table 11).

Secondary outcomes were as follows: One trial [56] reported statistically significant reductions in mean time to communicating the diagnosis to the patient and the mean time from test to treatment, but no effects on mean time from first contact to treatment (Table 12).

## Discussion

### Key Findings

We identified 42 controlled trials that investigated mobile technology-based interventions designed to improve health care service delivery processes. None of the trials were of high quality and nearly all were undertaken in high-income countries. Thirty-two of the trials tested interventions directed at health care providers. Of these trials, seven investigated interventions providing health care provider education, 18 investigated interventions supporting clinical diagnosis and treatment, and seven investigated interventions to facilitate communication between health care providers. None of the trials reported any objective clinical outcome, and the reported results for health care provider support interventions are mixed. There may be modest benefits in outcomes regarding correct clinical diagnosis and management delivered via application software, but there were mixed results for medical process outcomes regarding the time taken and completeness of or errors in reports or warning scores. For educational interventions for health care providers, there was no clear evidence of benefit. For interventions aiming to enhance communication between health care providers, one trial showed benefits in using the telephone functions of a mobile phone to enhance verbal communication between surgeons and nurses. Two trials showed reductions in the quality of clinical assessment using mobile technology based photos when compared to a gold standard and one trial reported a reduction in quality of ECG print outs delivered via mobile phones.

For the category of communication between health services and consumers, SMS reminders have modest benefits in increasing clinic attendance and appear similar in their effects to other forms of reminder. There is no evidence that SMS reminders influence appointment cancellations, but the 95% CIs for the pooled effect were wide. One trial [56] reported mixed results relating to time to treatment using SMS to notify patients of their test results.

### Strengths and Limitations of the Review

To our knowledge, this is the first comprehensive systematic review of trials of all mobile technology interventions delivered to health care providers and for health services support to improve health or health services. The review expands and updates the findings of earlier systematic reviews that focussed on specific devices, and/or specific functions, and/or specific health topics [8],[11],[58]. We identified more than twice the number of trials of educational interventions and trials of PDA applications identified in previous reviews [11],[58]. Our review findings are consistent with those of Krishna et al. and Car that text messages can reduce missed appointments [8],[59].

Our systematic review was broad in its scope. We only pooled outcomes where the intervention function (e.g., SMS messages), trial aim, and outcomes used in trials were the same. Here, findings in relation to clinical diagnosis and management and educational interventions are summarised, the individual trial results are reported in Tables 1–12. It was not appropriate to pool these results as the interventions targeted different diseases and outcomes. Further, there are likely to be important differences in the intervention content of these interventions (such as the behaviour change techniques used), even in those using the same mobile technology functions (such as application software). It was not possible to explore how different intervention components influenced outcomes as the intervention components were not described consistently or in detail in the authors' papers. It was not possible to explore how the intervention components targeting the disease and outcomes influenced the results.

It was beyond the scope of our review to review internet or video-based interventions not specifically designed for mobile technologies. We also excluded interventions combining mobile technologies with other interventions such as face-to-face counselling, which should be subject to a separate systematic review. Thirteen trials (31%) did not provide sufficient data to calculate effect estimates and authors did not respond to requests for data, which could have resulted in bias in the systematic review findings. Factors influencing heterogeneity of effect estimates include low trial quality, in particular inadequate allocation concealment [60], participant factors such as demographics or disease status, the setting (hospital, primary care), the intervention features (components, intensity, timing), the type of mobile technology device (e.g., PDA or mobile phone) or function (e.g., SMS, application software), and the nature of the control group (e.g., standard care in a high-income country or in a low-income country). We were unable to statistically explore factors influencing heterogeneity because there were few trials of similar interventions reporting the same outcomes, resulting in limited power for such analyses. It was not possible to statistically explore the mechanism of action of the interventions because there were too few similar interventions reporting the same outcomes. In addition, authors' descriptions of interventions were insufficiently detailed to allow mechanisms of action to be explored. It was outside the scope of this review to explore the cost-effectiveness of interventions with modest benefits such as appointment reminders.

At the request of the editors we re-ran our search on 1 November 2012 to any identify other trials eligible for this review published since our last search, and we identified eight trials. One high quality trial demonstrated that text message reminders increased Kenyan health workers' adherence to malaria treatment guidelines with improvements in artemether-lumefantrine management of 23.7 percentage points (95% CI 7.6–40.0) and immediate intervention of 24.5 percentage points (95% CI 8.1–41.0) and 6 mo [61]. Three trials reported statistically significant increases in clinic attendance with text message reminders (OR 1.61 [95% CI 1.03–2.51], respectively) [62]–[64]. These findings are similar to those reported in trials already included in the review [47]–[54]. One trial reported statistically significantly increased attendance with voice reminders compared to text message reminders [65]. One trial showed no effect on HIV viral load of a mobile phone-based AIDS care support intervention for community-based peer health workers [66]. One trial reported better performance in a cardiac arrest simulation for health care providers allocated to receiving a mobile phone application regarding advanced life support [67]. One trial reported more errors in interpreting ECGs delivered by MMS compared to paper print-outs [68].

### Meaning of the Study, Mechanisms of Action, Implications for Health Care Providers, or Policy

Trials of heath care provider support show some promising results for clinical management, appropriate testing, referral, screening, diagnosis, treatment, and triage. However, trials included in our review were subject to high or unclear risk of bias. In particular, only one of the 17 trials clearly reported that allocation was concealed and where there is no allocation concealment, the reported results may be an over-estimate of effects. To date no trials have reported effects of mobile technology-based clinical diagnosis and management support on objective health outcomes. Most of the trials supporting health care providers in clinical diagnosis and management employed PDA devices and customised application software functions. While PDA devices are no longer widely used, customised application software functions are now deliverable on smart phones or tablets. Mobile technology-based interventions may not be suitable for some clinical processes.

The data available for making clinical diagnoses or calculating early warning scores may be reduced and the time taken for medical processes may be increased. There was no clear evidence of benefit of mobile technology-based educational interventions for health care professionals. For interventions using mobile technologies to communicate visual data, there were increases in time to diagnosis or ECG transmission or diagnostic errors. Two trials using photos taken by mobile phone reduced diagnostic accuracy of fractures, skin ecchymoses, and potential to perform re-implantation when compared to a gold standard. However, the use of such technologies may be more relevant for settings where the gold standard is not available. Furthermore, the quality of photos on mobile phones has improved since these studies were completed.

Mobile technology-based diagnosis and management support may be most relevant to health care providers in developing countries where mobile phones potentially allow clinical support and evidence-based guidance to be delivered to health care professionals working remotely and in circumstances where senior health care professional support or other infrastructure is lacking [69]. One high quality trial has reported increased adherence to malaria treatment guidelines by health care workers in Kenya [61]; however, the evidence from controlled trials to date is mostly from high-income countries where the control group “standard care” may be very different to “standard care” available in low- or middle-income countries.

SMS messages are modestly effective as appointment reminders. Their effects appear similar to other forms of reminder. Health care providers should consider implementing SMS appointment reminders because the cost of missed appointments in health services is high, the cost of providing SMS appointment reminders is low, and SMS reminders are cheaper than other forms of reminder (e.g., a letter with stamp).

### Unanswered Questions and Future Research

High quality trials should be conducted to establish the effects of clinical diagnosis and management support (such as protocols/decision support systems) on clinical outcomes using customised application software functions on mobile phones. The effects of such support on the management of different diseases and on objective disease outcomes should be evaluated. It is imperative that future trials of clinical decision support, guidance, and protocol delivered via mobile technologies take place in low- and middle-income countries. Many of the interventions evaluated to date are single component interventions of low intensity. The effects of higher intensity multi-component mobile technology interventions should be evaluated. Authors must describe the components of future interventions in detail so that mechanisms of action and the impact of different components on outcome can be explored.

Trials should evaluate the effects of the use of photographic or video functions to support health care providers compared to standard care (where gold standard options are not available). As the technological capabilities of mobile phones improve, such as in photographic quality, further trials of the effects of using photos taken on mobile technologies on diagnostic accuracy may be a warranted. Further research should evaluate the effects and cost-effectiveness of mobile technologies to increase the speed of communication between clinicians and patients, such as test results.

Interventions combining elements delivered by mobile technology with other treatments such as clinics based counselling combined with text messages should be systematically reviewed.

### Conclusion

The reported effects of health care provider support interventions are mixed. Trials report modest benefits for clinical diagnosis and management support outcomes. For interventions for health services, SMS reminders have modest benefits on attendance. Service providers should consider implementing SMS appointment reminders. One high quality trial published since our literature search was completed shows benefits in adherence to malaria treatment guidelines [61]. In other areas, high quality trials are needed to robustly establish the effects of optimised mobile health care provider interventions on clinically important outcomes in the long term.

## Supporting Information

Checklist S1 **PRISMA checklist.**
(DOC)

Table S1 **Excluded studies.**
(DOCX)

Text S1 **Search strategy.**
(DOCX)

Text S2 **Systematic review protocol.**
(PDF)

## Abbreviations

ECG: electrocardiogram
m-Health: mobile-health
MMS: multimedia message
PDA: personal digital assistant
RR: relative risk
SMS: short message service
